# TTP-like syndrome and its relationship with complement activation in critically ill patients with COVID-19: A cross-sectional study

**DOI:** 10.1016/j.heliyon.2023.e17370

**Published:** 2023-06-16

**Authors:** Mohammadreza Ardalan, Mohammadreza Moslemi, Azin Pakmehr, Sepideh Zununi Vahed, Amirreza Khalaji, Hamidreza Moslemi, Amir Vahedi

**Affiliations:** a Kidney Research Center, Tabriz University of Medical Sciences, Tabriz, Iran; b Department of Internal Medicine, School of Medicine, Tabriz University of Medical Sciences, Tabriz, Iran; c Department of Oral and Maxillofacial Surgery, Dental School, Shahid Beheshti University of Medical Sciences, Tehran, Iran; d Department of Pathology, Tabriz University of Medical Sciences, Tabriz, Iran

**Keywords:** Thrombotic microangiopathies, Thrombotic thrombocytopenic purpura, COVID-19, Complement system

## Abstract

**Background:**

The covid-19 disease has caused many deaths worldwide since December 2019. Many thromboembolic events, such as VTE and TTP, have been reported since the beginning of this pandemic. Considering the prominent role of complement in developing TTP and TTP-like syndrome in recent studies, this study aimed to assess the prevalence of TTP-like syndrome and its relationship with complement activity in critically ill patients with COVID-19.

**Method:**

This study was conducted on 77 COVID-19 patients admitted to the ICU wards of Tabriz Imam Reza hospital from March to June 2021. TTP-like syndrome was diagnosed using a blood specimen for evidence of thrombocytopenia, microangiopathic hemolysis (low hemoglobin, increased LDH level, schistocytes in a peripheral blood smear, and negative direct agglutination test), and end-organ injury, including acute kidney injury or neurological deficit. ADAMTS 13 activity levels could not be achieved owing to logistic issues; therefore, we could not accurately diagnose TTP and TTP-like syndrome based on ADAMTS 13 levels, so to increase the accuracy of diagnosis, we have included people with classical pentad evidence in the TTP-like syndrome group. Complement parameters, including C3, C4, and CH50, were measured.

**Result:**

Seven cases of TTP-like syndrome were diagnosed using the previously mentioned criteria, which stands for 9.1% of the study population. Compared with patients without TTP-like syndrome, C3 was significantly lower in patients with TTP-like syndrome (p-value = 0.014), and C4 and CH50 demonstrated insignificant differences between the two groups (p-value = 0.46, p-value = 0.75).

**Conclusion:**

Our study showed that the TTP-like syndrome was present in a significant percentage of critically ill patients with COVID-19. Lower C3 levels in TTP-like syndrome-diagnosed patients can indicate complement activation as one of the influential factors in initiating TTP-like syndrome in COVID-19 patients. More studies are recommended to clarify the exact mechanism to achieve adequate therapeutic methods and better manage the disease and its complications.

## Highlights

• TTP-like syndrome was present in a significant percentage of critically ill patients with COVID-19.
• These studies agree with observations that complement activation is involved in ARDS (acute respiratory distress syndrome) and Organ Dysfunction Syndrome (MODS) pathophysiology.
• The lower C3 level in patients with TTP-like syndrome can indicate the critical role of complement pathway activation in this phenomenon.

## 1 Introduction

Coronavirus disease 2019 (COVID-19), caused by severe acute respiratory syndrome coronavirus 2 (SARS-CoV-2), has become a catastrophic health crisis since December 2019 [1]. The disease caused 3.5 million deaths worldwide in 2021 and its marching worldwide is continuing [2]. COVID-19 was soon discovered to affect multiple systems, including the heart, kidney, liver, coagulation, and respiratory system [3].

As the pandemic spread worldwide, more studies have shown a positive correlation between COVID-19 and coagulopathies, such as deep vein thrombosis, pulmonary thromboembolism, and thrombotic microangiopathies [4,5]. Severe COVID-19 was associated with thrombotic microangiopathy, complement activation, and endotheliopathy [6,7,8]. Thrombotic microangiopathies (TMA) is a broad pathophysiologic process used to describe the occlusive microvascular or macrovascular disease, often with intraluminal thrombus formation, but is also defined clinically by microangiopathic hemolytic anemia and thrombocytopenia (MAHAT), and organ damage [9]. The most common type of TMA is thrombotic thrombocytopenic purpura (TTP), a systemic disorder of microvascular thromboses due to deficiency or inactivation of ADAMTS-13 [9]. Thrombotic thrombocytopenic purpura was characterized initially by TMA, signs of neurological deficit, and renal impairment. The diagnosis is confirmed by ADAMTS13 activity level <10% but the emergency treatment decision must be made after the initial assessment (clinical history, examination, and routine laboratory parameters of the patient, including peripheral blood smear) [10].

TTP-like syndrome occurs with similar hematologic changes and other organ dysfunction syndromes with mild to moderate deficiency of ADAMTS13, about 25–75% of normal, and is more common in patients with critical illnesses and sepsis [11]. The final mechanism of both phenomena in the creation of micro thrombosis is disseminated intravascular micro thrombosis (DIT) and vascular microthrombotic disease. However, it seems that activation of the complement system, which produces a terminal complement complex, C5b-9plays an essential role in creating endotheliopathy and vascular microthrombotic disease in TTP-like syndrome [12].

Based on recent studies, it seems that endotheliopathy-associated vascular microthrombotic disease and complement activation play an essential role in the physiopathology of ARDS and Organ Dysfunction Syndrome (MODS) and Acute respiratory distress syndrome (ARDS) Covid-19 in critically ill patients [6,7,8,13,14].

This study aimed to examine the prevalence of TTP-like syndrome and the hypothesis of its relationship with complement activation in COVID-19 ICU admitted patients.

## 2 Patients and methods

### 2.1 Patients’ selection

This study was conducted on patients admitted at the ICU units of Imam Reza teaching hospital of Tabriz University of Medical Sciences, Iran, from March to June 2021. Patients met the following inclusion criteria: polymerase chain reaction positive test for SARS-CoV-2; pneumonia confirmed by Computed tomography (CT) of the chest; all patients had mild to severe ARDS according to Berlin ARDS criteria. All participants diagnosed with severe SARS-CoV-2-related pneumonia had a high risk of multiple organ injuries. Exclusion criteria were active malignancies and previous solid organ or bone marrow transplantation. The study was approved by the ethics committee of Tabriz University of Medical Sciences (Ethical code: IR. TBZMED.REC.1399.1107). Demographic characteristics were recorded, including age, gender, and history of diabetes mellitus and cardiovascular diseases.

**Fig. 1:**
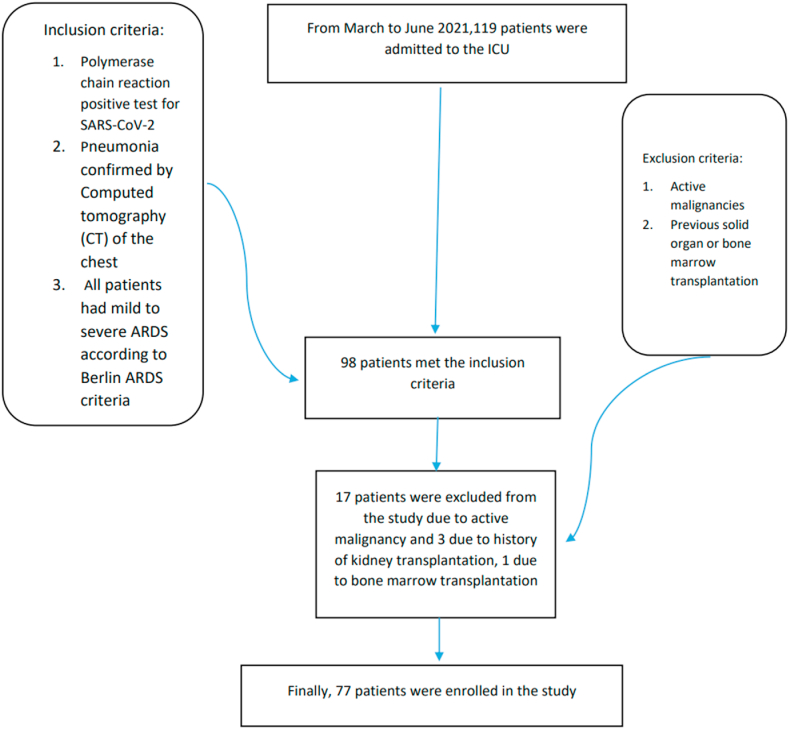
Patients' selection.

**Fig. 2:**
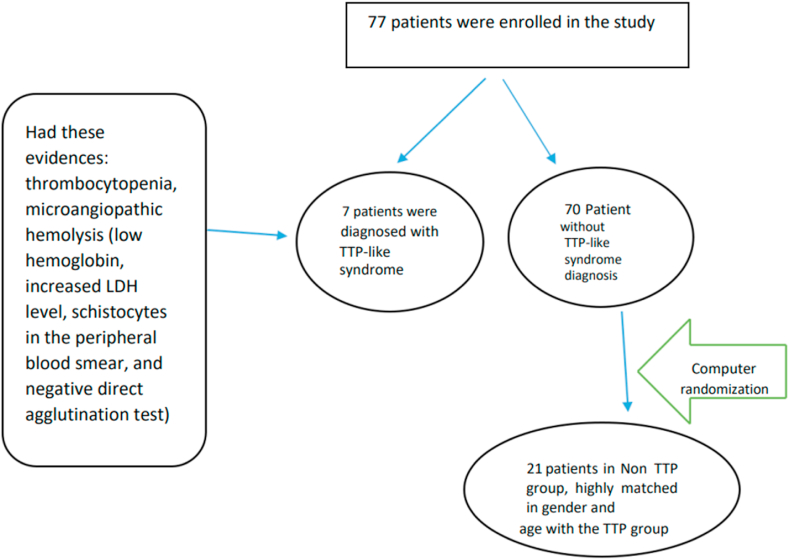
A group of patients was separated from the eligible population without TTP-like syndrome through computerized randomization to compare several characteristics of TTP and no TTP group.

From March to June 2021,119 patients were admitted to the ICU units organized for Covid-19 patients. 98 patients met the inclusion criteria. Among these, 17 patients were excluded from the study due to active malignancy and, 3 due to a history of kidney transplantation, 1 due to bone marrow transplantation. Finally, 77 patients were enrolled in the study (Fig. 1). A group of patients from the eligible population without TTP-like syndrome was selected via computer randomization using Research Randomizer software (Fig. 2) to compare several characteristics of the TTP-like syndrome group and no TTP-like syndrome group.

### 2.2 Measurement

TTP-like syndrome was diagnosed using blood specimens for evidence of thrombocytopenia, microangiopathic hemolysis (low hemoglobin, increased LDH level, schistocytes in the peripheral blood smear, and negative direct agglutination test), normal prothrombin time (PT), partial thromboplastin time (PTT), international normalized ratio (INR), and end-organ injury including increased creatinine and blood urea nitrogen. Moreover, neurological abnormalities such as headaches, seizures, and impaired mental states were observed and recorded. Measurements of ADAMTS 13 activity, VWF antigen, and Factor VIII levels could not be achieved owing to logistic issues; therefore, we could not accurately diagnose TTP and TTP-like syndrome based on ADAMTS 13 levels, so to increase the accuracy of diagnosis, we have included people with classical pentad evidence in the TTP-like syndrome group.

C3, C4, and CH50 molecule blood levels were measured using the Shenzen Genrui kit for C3 and C4 (Genrui; Genrui Biotech Inc., Shenzhen, China) and DKO040 - CH50 – IFU kit for CH50 (Diametra, Italy). Blood samples (EDTA-anticoagulated plasma, sodium-citrate anticoagulated plasma, and native serum) were taken by antecubital venipuncture or central catheter. All plasma samples were collected from the fifth to the ninth day of admission.

### 2.3 Statistical analysis

Qualitative variables are expressed as percentages, and quantitative variables are expressed as mean ± standard deviation. Data were analyzed by IBM SPSS statistics version 20. Independent Student *t*-test and Mann-Whitney were used to analyze quantitative variables and Chi-square was used to analyze qualitative ones. P < 0.05 was reflected to be statistically significant.

## 3 Result

This study was conducted on 77 patients, including 51 men and 26 women, whose mean age was 64.31 ± 17.52 years. More details on demographic characteristics are shown in Table 1.

**Table 1:** Demographic characteristics of the study population.

	Minimum	Maximum	Mean	Std. Deviation
Age (year)	23	93	64.31	17.521
Platelet (count)	8000	400000	174529.8701	87297.75283
Hemoglobin (mg\dl)	6.80	17.00	12.0234	2.71651
Reticulocyte (%)	0.5	3.50	1.2413	0.62082
LDH (Unit\Liter)	200.00	1600.00	830.5556	290.12707
INR	0.90	6.00	1.2157	0.74845
PT (second)	11.00	45.00	14.6883	5.27970
PTT (second)	30.00	70.00	41.6753	8.66852
Urea (mg\dl)	22.00	181.00	62.0390	35.66417
Creatinine(mg\dl)	0.50	3.98	1.3529	.65382
C3 (mg\dl)	0.75	2.82	1.7925	.44183
C4 (mg\dl)	15.00	79.00	26.0390	11.25359
CH50 (Unit\dl)	2.00	295.00	161.1429	56.17892

LDH: lactate dehydrogenase, PT: normal prothrombin time, PTT: partial thromboplastin time, INR: international normalized ratio.

Seven cases of TTP-like syndrome were diagnosed using the mentioned criteria, which stands for 9.1% of the study population. This group consisted of 4 women and three men with an average age of 56.14 ± 17.22 years. One patient had Diabetes mellitus (DM), two patients had a positive history of cardiovascular disease (CVD), and one suffered from both. The control group consisted of 21 patients from the study population without TTP-like syndrome who were highly matched in gender and age with the case group (p-value = 1 and p-value = 0.995, respectively). The levels of C3, C4, and CH50 were compared between this group and the TTP-like syndrome patients (Fig. 2). C3 was significantly lower in patients with TTP-like syndrome (p-value = 0.014), while C4 and CH50 demonstrated insignificant differences between the two groups (p-value = 0.46, p-value = 0.75), Table 2.

**Table 2:** The mean of complement parameters in COVID-19 patients with and without TTP-like syndrome.

Variables	TTP-like syndrome	No TTP-like syndrome	*P*-value
C3 (mg\dl)	1.28 ± 0.43	1.72 ± 0.36	0.014
C4 (mg\dl)	25.35 ± 0.21	24 ± 0.031	0.46
CH50 (Unit\dl)	142.71 ± 65.44	136.28 ± 39.87	0.75

## 4 Discussion

Our study diagnosed several TTP-like syndrome cases among 77 ICU-admitted COVID-19 patients, indicating the high prevalence of TTP in critically ill patients with COVID-19. Previously, multiple paper studies have described COVID-19 patients who developed TTP during their disease course [15,16,17]. Studies have shown that TMA plays a crucial role in the pathogenesis of ARDS and MODS in COVID-19 patients [18,19]. Several mechanisms have been hypothesized for microangiopathies in covid19 patients. Some proposed inhibitors of the ADAMTS13 as the primary mechanism of acquired TTP in COVID-19 patients [20,21,22]. Two other studies hypothesized that virus invasion of the vascular endothelium and endothelitis cause TTP [23,24]. Five cases of COVID-19 with complement-mediated microvascular injury were described by Cynthia Magro [13]. Evidence indicates that hyperactivation of complement is involved in the pathogenesis of microangiopathies and COVID-19 disease [23,25].

Jae C. Chang suggested that TTP-like syndrome is a more common type of TMA in critically ill patients with COVID-19. TTP-like syndrome is a hemostatic disorder in critical illnesses, but TTP occurs as a hereditary or autoimmune disease. In TTP, microthrombi get stuck in the brain and kidney microvasculature, while in TTP-like syndrome, they tend to get stuck in liver and lung vessels and the brain and kidney. ADAMTS13 deficiency causes the accumulation of circulating ultra-large von Willebrand factor (ULVWF), which can trigger microthrombogenesis and TTP. Unlike TTP, TTP-like syndrome occurs in critical illnesses due to complement activation. In response to viral septicemia, the immune system activates the complement cascade, which produces terminal complement complex C5b-9. This can induce endotheliopathy, which triggers inflammatory and microthrombotic pathways—both pathways lead to platelet activation, and exocytosis of ULVWF from endothelial cells causes microthrombosis [26].

Our findings demonstrated a significantly lower amount of C3 in patients with TTP-like syndrome than in the control group without TTP-like syndrome. This result was confirmatory in accordance with the study of de Nooijer AH et al. This study showed that compared with healthy controls, complement factors C3a, C3c, and terminal complement complex were significantly increased in the plasma of patients with COVID-19. They reveal that these complement factors were elevated considerably in intensive care unit patients during the entire disease course [6].

Mechanistically, SARS-CoV-2 can activate the complement system either directly (by activating classical, lectin, or alternative pathways) or indirectly by causing endothelial cell damage and dysfunction (endotheliopathy) and thromboinflammation. Envelope proteins of SARS-CoV-2 can bind to mannose-binding lectin (MBL) and activate the lectin pathway or initiate the classical pathway through C1q and virus-specific antibodies [27,28].

Moreover, viral spike protein may bind to heparin sulfate and compete with factor H (a negative regulator of complement activity), consequently, dysregulating alternative complement pathways [29]. These studies do agree with observations indicating that complement activation is involved in the pathophysiology of ARDS [30,31]. Correspondingly, high levels of soluble C5b–9 and C5a in the circulation of cases with severe COVID-19 were correlated with clinical severity [32,33]. Furthermore, C3 overactivation and consumption (decrease in C3 concentrations and elevated ratio of C3a/C3) were identified as significant risk factors for COVID-19-related mortality [34].

Lectin pathway activation of complement in response to SARS-CoV-2 infection potentiate the adhesion of the inflammatory cells into endothelial cells and induces the production of inflammatory factors and local formation of NETs (neutrophil extracellular traps), leading to platelet activation and endotheliopathy [27].

The diagnosis of TTP is confirmed by ADAMTS-13 activity <10% [9]. ADAMTS-13 activity in TTP-like syndrome is more than 10% [11]. The main limitation of the present study was the need for more facilities to measure the level of ADAMTS13 and its inhibitor in the country, which made it impossible to diagnose TTP and TTP-like syndrome based on ADAMTS 13 levels.

In this study, we could compare several characteristics of both groups and demonstrate the significantly lower amount of C3 in patients with TTP-like syndrome. Also, the larger population would have made the study more accurate.

To conclude, the present study demonstrated that the complement system is also activated in critically ill patients with COVID-19 admitted to the ICU. Moreover, our study suggests an essential role of the complement system in the pathophysiology of TTP-like syndrome in these patients.

More studies are recommended to clarify the exact mechanism to achieve adequate therapeutic methods and better manage the disease.

## Author contribution statement

Mohammadreza Ardalan: Conceived and designed the experiments.

Mohammadreza Moslemi: Conceived and designed the experiments, Performed the experiments, Wrote the paper.

Azin Pakmehr: Performed the experiments, Wrote the paper.

Sepideh Zununi Vahed and Amirreza Khalaji: Contributed reagents, materials, analysis tools or data.

Hamidreza Moslemi: Analyzed and interpreted the data.

Amir Vahedi: Performed the experiments.

## Data availability statement

Data will be made available on request.

## Declaration of competing interest

The authors declare that they have no known competing financial interests or personal relationships that could have appeared to influence the work reported in this paper.
